# Reliability and preliminary reference values for the Total Faulty Breathing Scale (TFBS): A cross‐sectional study

**DOI:** 10.1002/hsr2.661

**Published:** 2022-05-24

**Authors:** Vikram Mohan, Jonathan Branney, Carol Clark, Rebecca Neal, Madhanagopal Jagannathan, Aatit Paungmali, Maria Perri

**Affiliations:** ^1^ Department of Rehabilitation and Sports Sciences, Faculty of Health and Social Sciences Bournemouth University Bournemouth UK; ^2^ Department of Nursing Science, Faculty of Health and Social Sciences Bournemouth University Bournemouth UK; ^3^ Healthy Ageing Physiotherapy and Research Centre Coimbatore Tamil Nadu India; ^4^ Neuro‐Musculoskeletal and Pain Research Unit, Department of Physical Therapy, Faculty of Associated Medical Sciences Chiang Mai University Chiang Mai Thailand; ^5^ Perri Chiropractic, Wellness Springs Highland Mills New York USA

**Keywords:** breathing function, interrater reliability, intrarater reliability, scale

## Abstract

**Background and Aims:**

The evaluation of breathing function is crucial in the clinical examination of the respiratory system. The Total Faulty Breathing Scale (TFBS) could be used in clinical settings to quantify the measurement of breathing dysfunction. Reliability data for the TFBS are available for males, but there is a requirement to determine reliability for females and to develop reference values. The aim of this study, therefore, was to determine the reliability in females and to establish the preliminary reference values for the TFBS.

**Methods:**

Twenty‐three healthy female participants for reliability and 44 (7 male and 37 female) participants for preliminary reference values participated in this cross‐sectional study. For both aspects of the study, participants were instructed to breathe at their own pace with no specific instruction. Then each participant was observed carrying out normal breathing for a period of 10 breaths and deep breathing for a period of 10 breaths while being assessed with the TFBS.

**Results:**

Intrarater and interrater reliability of the TFBS showed a kappa value of 0.769 and 0.751, respectively, indicating substantial agreement for female participants. The preliminary reference categories for TFBS were reported to be normal for 20 (45.5%) participants and mild faulty breathing for the remaining 24 (54.4%) participants.

**Conclusions:**

The findings of this study suggested that TFBS was reliable to measure breathing function among female participants, and the reference categories may be helpful in the identification of normal and faulty breathing.

## 1 INTRODUCTION

Breathing consists of mechanical, physiological, and psychological processes, any one of which, if faulty, can cause breathing problems.^1^, ^2^, ^3^ The diaphragm, intercostal muscle, and abdominal muscles comprise the respiratory muscles. Normal and quiet inspiration descends the diaphragm along with external intercostal muscles.^4^ The action of external intercostal muscles elevates the rib cage during quiet breathing and the accessory muscle helps in forced breathing. Changes in breathing mechanics lead to reduced lung volume and impaired ventilation and perfusion ratios.^5^ This affects both oxygen and carbon dioxide concentrations in the blood. In cases of mild and moderate mechanical dysfunction, this may impact a person's ability to walk, whereas severe dysfunction can result in significant disability, complete respiratory failure, and death. The prevalence of people with dysfunctional breathing is thought to be approximately 9.5% in the United Kingdom among the normal population.^6^ Changes in breathing mechanics can be treated effectively with therapeutic exercises aimed at changing breathing mechanics and reducing the negative effects of muscular imbalances, motor control alterations, and physiological adaptations.^7^

Tools that can be used for assessing anterior−posterior chest diameter and breathing patterns include the respiratory movement measuring instrument (RMMI) and respiratory inductive plethysmography (RIP).^8^, ^9^, ^10^ The intrarater reliability of RMMI has been found to be moderate to strong (*r* = 0.54−0.94) and interrater reliability was strong (*r* = 0.71−0.99) except for the left thoracic position.^11^ The reliability study on RMMI included correlation statistics only, and hence a more precise intrarater and interrater reliability for agreement is unknown. The tools which use physical examination for assessing dysfunctional breathing are Hi Lo breathing and manual assessment of respiratory motion (MARM), and the reliability was established for various measures of MARM.^12^, ^13^ Even though reliability and validity are established for certain tools, these are costly tools, take time to complete, and are not readily available in clinical practice, especially in primary care.^8^, ^14^ Therefore, there is a requirement to develop simple measures that are easy to use in clinical practice.

The Total Faulty Breathing Scale (TFBS) was developed to identify dysfunctional breathing and is used to assess breathing in both quiet and deep breathing.^15^ The purpose of the TFBS scale grading is to differentiate between normal and abnormal breathing patterns accurately and consistently. The TFBS could be used to objectively describe the extent of dysfunctional breathing, which is not readily apparent without close observation. The scale evaluates the participants according to physical signs and may be relevant to pulmonary disease.^16^ It uses observations of the absence of lateral rib motion, lifting of the clavicle, and paradoxical breathing, which are categorized on a scale of normal (0), mild (1−4), moderate (5−8), and severe (9−12).^15^ An earlier reliability study of the TFBS was undertaken with male participants and demonstrated percent agreement of 96% for both intra‐ and interrater reliability with a kappa score of 0.78, indicating substantial agreement.^15^ However, currently, there are no studies on the reliability of TFBS among female participants and no published reference values for the TFBS. In addition, the development of preliminary reference values is needed for healthy participants using TFBS before reference values can be determined in clinical populations. Hence, there is a requirement to explore the reliability of the tool in female participants and to develop a preliminary reference value that may be helpful in the identification of abnormal breathing patterns. The aim of the study was to explore the intrarater and interrater reliability of female subjects and to identify preliminary reference values using TFBS.

## 2 METHODS

This was a cross‐sectional study design that followed the strengthening of the reporting of observational studies in epidemiology (STROBE) statement.^17^ The convenience sample was composed of female volunteers 18 years or older for the reliability study, and for reference values the participants were male and female and aged between 18 and 24 years old. The participants were excluded if they had a history of medical, neurological, or musculoskeletal impairments that could affect the breathing measurement. Participants were assessed in a university‐based laboratory setting from July 1, 2020 to November 30, 2021.

A total of 27 female participants were required to establish *α* = 0.05 and *β* = 0.20, when the one‐way random effects model was used for estimating reliability, as described by earlier guidelines and is also based on previous work for the male subjects.^18^ For the preliminary reference values, we recruited 44 participants. Ethical approval was obtained through the university ethics committee (Ref. No. 31534) and before data collection, informed consent was obtained from each participant.

### 2.1 TFBS

The TFBS uses a range of values to differentiate between normal, mild, moderate, and severe breathing patterns. The grading of normal and dysfunctional breathing in the scoring system is as follows: normal: 0, mild: 1−4, moderate: 5−8, and severe: 9−12.

Initially, the subjects were instructed to breathe at their own pace with no other specific instruction. Then, each participant was observed carrying out normal breathing for a period of 10 breaths and deep breathing for a period of 10 breaths. All measurements were taken within 2−3 m between the researcher and participant to mirror clinical practice following COVID‐19 infection control policy measures. The breathing assessment was carried out in an upright standing position against a white background and the data were recorded for reliability and reference values. The presence or absence of faulty breathing was the outcome variable, assessed by observation of the clavicle, lateral rib, and abdominal movement. In clinical situations, the examiner would base a diagnosis on history and on more thorough physical observations, and clinical investigations and tests as indicated.

### 2.2 Reliability

Independently from one another, one male physiotherapist with 20 years of experience and a male registered nurse with 20 years' experience simultaneously evaluated the participants in this study. The TFBS was conducted across 2 days, with an interval of 7−28 days between repeat measurements. Participants were evaluated at the same time of day to avoid diurnal variations.

### 2.3 Reference values

A research assistant who was a final year physiotherapy student collected data for the reference value for a period of 3 months and then one of the investigators carried out the data collection for the reference values.

### 2.4 Statistical analysis

Anonymized data were exported from Microsoft Excel spreadsheet into SPSS (version 28) for analysis. Data and analyses are reported in line with the statistical analyses and methods in the published literature (SAMPL) guidelines.^19^ Descriptive statistics such as distribution of age, height, and weight are presented as mean and standard deviation (SD). Similarly, the descriptive statistics for TFBS were presented as frequency and percentage as these values present the reference values for the age group selected. Evaluation of intrarater reliability and interrater reliability of the assessment of normal and faulty breathing patterns were determined using percent agreement statistics and kappa statistics. Kappa statistics was used as this is considered a robust measure to assess reliability.^20^ The interpretation of kappa value was made based on guidelines as follows: “<0” indicated less than chance agreement, “0.01−0.20” indicated slight agreement, “0.21−0.40” indicated fair agreement, “0.41−0.60” indicated moderate agreement, “0.61−0.80” indicated substantial agreement, “0.81−0.99” indicated almost perfect agreement and “1.00” indicated perfect agreement.^21^ The statistical significance was set at the alpha level of 0.05.

## 3 RESULTS

### 3.1 Reliability

Twenty‐three female individuals comprised the sample with a mean (SD) age of 29 (9.1) years, height 166 (5.4) cm, and weight 73 (15.7) kg. Table 1 shows the intrarater reliability and interrater reliability when assessing breathing patterns in females. The results of the percent agreement for the TFBS are more than 80% and the kappa score was 0.77, which showed substantial intrarater agreement for Examiner 1. The percent agreement for the TFBS between the examiners was more than 85% and the kappa score was 0.75, which also displayed substantial agreement.

**Table 1:** Intrarater and Interrater reliability of using the Total Faulty Breathing Scale

Examiners	Percent agreement	Kappa score	Standard error	*p* Value
Interrater (*n* = 23)	87	0.751	0.128	<0.001
Intrarater (Examiner 1) (*n* = 18)	83.3	0.769	0.150	<0.001

### 3.2 Reference values

Descriptive statistics for the study participants and the reference values of the TFBS scale are presented in Table 2. Out of 44 participants selected for the reference values, 24 participants showed mild faulty breathing scores and the remaining 20 participants showed a normal breathing score.

**Table 2:** Descriptive statistics of the TFBS (reference value)

Variables	Mean (SD)/Frequency (%) [*n* = 44]
Age (years)	20 (1.8)
Gender	
Male	7 (15.9%)
Female	37 (84.1%)
Height (cm)	169 (7.5)
Weight (kg)	65.9 (9.5)
TFBS	
Normal	20 (45.5%)
Mild	24 (54.4%)

Abbreviations: cm, centimeters; kg, kilograms; TFBS, Total Faulty Breathing Scale.

## 4 DISCUSSION

The present study was undertaken to evaluate the reliability of the TFBS. An advantage of this scale in comparison to previously described methods is that it only takes less than 5 min to complete and may not need specialized training.^12^, ^13^

Our results show that the two experienced healthcare professionals were able to reach a substantial agreement on the breathing scores of female participants. The study results were in accordance with an earlier study that was carried out on male participants, which showed substantial agreement for one examiner and almost perfect agreement for the second examiner.^15^

In the present study, care was taken to develop a standardized observation procedure that can be applied in clinical practice. Initially, the assessors conferred on the assessment criteria. For instance, it was agreed that “lifting of clavicle” needed to be accompanied by increased work of neck muscles to score this as two for both quiet and deep breathing. No further attempts were made to minimize the assessor variance or for the grading.

As can be expected, the intrarater agreement on the breathing measurement could be higher than the agreement between the examiners, but in this study the interrater percent agreement is more than 85%. This could be due to the number of participants who took part in the study (Table 1). However, the kappa score is also accounted along with percent agreement and the kappa score is smaller, which is expected for interrater agreement. Both raters agree well on the normal and mild categories of TFBS. For the normal category, there was only 8.7% difference and for the mild faulty breathing score, there was only 4.4%. However, the agreement for one of the participant's scores was moderate, which was not agreed between the raters, which fell in the category on a score between 4 and 5. Hence, this could be attributed that the difference in score is not the most obvious between the raters. Therefore, when it comes to categorizing the score was between mild (1−4) to moderate (5−8). On the other hand, the 11% difference was observed within the rater for normal and for the mild faulty breathing score. On associating, the reference values, this could be further inferred almost 45% participants were normal and the remaining 54% were having a mild faulty breathing score. From these categories, it can be interpreted that almost all the participants demonstrated between normal and mild faulty breathing patterns.

TFBS acts as a reliable tool and identifies faulty breathing by identification of the absence of outward lateral rib motion, lifting of the clavicle, and paradoxical movement, which is common in diseased populations.^16^ Similarly, a score of zero signifies the presence of outward lateral rib motion, no movement of the clavicle, and normal thoraco‐abdominal breathing in healthy subjects. Therefore, from the study results, it was determined that the TFBS is a reliable tool for assessing normal and dysfunctional breathing even in healthy subjects. When the scale is applied to the clinical population with pathology, this may have the potential to categorize the severity of dysfunctional breathing. In addition, it may have utility in monitoring response to treatment.

### 4.1 Limitations

The study used a convenience sample and therefore does not represent the entire population. There were five dropouts for the second visit, due to personal reasons or isolation from COVID‐19. Moreover, due to nationwide Covid‐19 access restrictions, only those who were permitted to attend campus for limited teaching activities were recruited, resulting in a smaller sample size. In addition, the test−retest reliability was conducted over 2 days, with an interval of at least 7 and at most 28 days as we were unable to recruit the participant in limited time duration; this could be attributed due to participants' availability and other measures in place as because of the Covid‐19 pandemic.

### 4.2 Perspective/recommendations

Assessing breathing with a TFBS shows great potential as a reliable clinical tool for evaluating faulty breathing. Furthermore, it is an easy tool to use, making it ideal for the clinical setting. Historically, the observational method is used to assess different types of breathing patterns.^22^ However, clinically, the TFBS tool offers an advantage of scoring breathing patterns by observation method. Further studies concerning the breathing pattern assessment should be carried out in subjects with musculo‐skeletal and cardio‐respiratory problems, to investigate the applicability in this population. In addition, the assessor needs to be knowledgeable of the mechanics of normal breathing and abnormal breathing pattern before utilizing the tool. Consideration of future studies to perform validity by comparing TFBS with other potential measures for assessing breathing function is needed.

### 4.3 Conclusion

The findings of this study indicate that the TFBS tool detects both normal and faulty breathing in asymptomatic female participants and can provide the clinician with a method to quantify the breathing pattern. The TFBS tool used in this study can be easily and efficiently used in a clinical setting. In addition, preliminary reference values of healthy individuals are provided.

## AUTHOR CONTRIBUTIONS

**Vikram Mohan**: Conceptualization; data curation; formal analysis; writing—original draft; writing—review and editing. **Jonathan Branney**: Conceptualization; data curation; writing—review and editing. **Carol Clark**: Conceptualization; writing—review and editing. **Rebecca Neal**: Conceptualization; writing—review and editing. **Madhanagopal Jagannathan**: Conceptualization; writing—review and editing. **Aatit Paungmali**: Conceptualization; writing—review and editing. **Maria Perri**: Conceptualization; writing—review and editing.

## CONFLICTS OF INTEREST

The authors declare no conflicts of interest.

## TRANSPARENCY STATEMENT

The manuscript is an honest, accurate, and transparent account of the study being reported; that no important aspects of the study have been omitted.
